# Comparison of two close‐packing methods for vertex placement in lattice radiotherapy (LRT)

**DOI:** 10.1002/acm2.70850

**Published:** 2026-10-21

**Authors:** Yingjie Song, Pan Ma, Jianrong Dai

**Affiliations:** ^1^ Department of Radiation Oncology National Cancer Center National Clinical Research Center for Cancer Cancer Hospital Chinese Academy of Medical Sciences and Peking Union Medical College Beijing China

**Keywords:** close‐packing arrangements, face‐centered cubic (FCC), hexagonal close‐packed (HCP), lattice radiation therapy (LRT), peak‐to‐valley dose ratio (PVDR), vertices arrangement

## Abstract

**Background:**

Lattice Radiation Therapy (LRT) is characterized by its 3D‐mouldated spatial dose distribution of vertices inside the lattice volume with high peak‐to‐valley dose ratio (PVDR) for enhancing tumor control and reducing normal tissue toxicity. While face‐centered cubic (FCC) and hexagonal close‐packed (HCP), both achieving maximum spatial packing density, have been used for vertex placement, a direct comparative analysis of their dosimetric performance is lacking.

**Purpose:**

This study aimed to systematically compare FCC and HCP methods for vertex placement under identical conditions to determine which yields superior dosimetric outcomes, specifically regarding the peak‐to‐valley dose ratio (PVDR), and to provide data supporting the standardization of LRT planning.

**Materials and methods:**

This study was retrospectively conducted on 10 patients with large gross tumor volumes (GTV) (GTV; median [IQR] GTV volume: 916.59[609.37] cm^3^). For each patient, an identical inward margin was applied to the GTV to create the lattice volume for placing LRT vertices. Within this lattice volume, two sets of spherical vertices were generated: one arranged in a HCP lattice and the other in a FCC lattice, both vertices of same diameter. The initial diameter and volume of vertices were determined based on GTV volume. Vertex positions were optimized using an Adaptive Simulated Annealing (ASA) algorithm to maximize inter‐vertex distance, with the total volume of all vertices constrained to approximately 1% of the GTV. All plans were optimized using identical dose‐volume objectives. Plan quality was evaluated using GTV dose metrics (D_5_%, D_10_%, D_50_%, D_90_%, D_mean_, V_50_%), normal tissue mean dose (NT D_mean_), maximum point doses (D_max_) to serial organs at risk and PVDR defined by both GTVD_10_/D_90_ and lattice volume D_10_/D_90_ ratios.

**Results:**

Vertex parameters (number, volume, spacing) showed no significant difference between Plan_FCC and Plan_HCP (*P* > 0.05). Dosimetric analysis revealed a statistically significant increase in PVDR (lattice volume D_10_/D_90_) for Plan_FCC compared to Plan_HCP (median [IQR]: 6.95 [3.38] vs. 4.48 [2.00], *p* = 0.019). No other evaluated metrics demonstrated significant differences, including other GTV metrics NT Dmean or the maximum point doses to serial OARs.

**Conclusions:**

Under matched radiation delivery technique, geometric constraints and identical dose‐volume objectives, the FCC vertex arrangement yields a statistically significantly higher peak‐to‐valley dose ratio (lattice volume D_10_/D_90_), compared to the HCP arrangement, without compromising other dosimetric indices. This finding suggests that FCC may be a preferred starting configuration for LRT vertex placement in large tumors and we recommend that the FCC arrangement be prioritized in clinical practice.

## 1 INTRODUCTION

LRT is characterized by creating three‐dimensional spherical high‐dose regions (vertices) within the lattice volume, generating a peak‐and‐valley dose distribution.^1^ As a form of spatially fractionated radiation therapy (SFRT), LRT mediates tumor cell kill and normal tissue protection through several interconnected radiobiological mechanisms. The highly heterogeneous dose distribution characteristic of SFRT triggers a cascade of responses, including bystander effects, abscopal effects, alterations in the microvascular network, and the induction of both local and systemic immune responses.^2^, ^3^ Wu et al.^1^ proposed that a high peak dose (∼18 Gy) with a rapid fall‐off to a low valley dose (20%–50% of the peak) is the key physical signature responsible for triggering the bystander and abscopal effects. This profound dose heterogeneity is considered the central mechanism that initiates antitumor immunity and enhances tumor control.^4^, ^5^

The optimal configuration of target vertices remains a pivotal consideration in the clinical investigation of LRT. The center‐to‐center distance (e.g., ∼2 cm) directly influences the size of the low‐dose region, consequently affecting the survival and activation of immune cells.^1^ Furthermore, the peak‐to‐valley dose differential is primarily governed by the number of vertices, the size of the beam, or the volume of each vertex.^6^ Based on an analysis of prior clinical LRT experiences, Wu et al.^3^, ^7^ have consolidated a widely adopted set of technical recommendations for clinical LRT implementation, as illustrated in Figure 1. This framework specifies parameters for vertex location, size, spacing, and quantity: vertices are typically positioned inside the lattice volume, which maintains a distance of 0.5–1.5 cm from the GTV boundary to avoid excessive irradiation of surrounding normal tissues. The center‐to‐center distance between vertices is recommended to be 2–5 cm, with individual vertex diameters ranging from 0.5 to 3 cm. The volume of vertices is typically recommended to constitute 1%–10% of the GTV volume.

**FIGURE 1:**
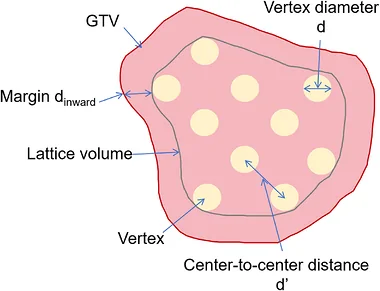
Typical plan pattern of lattice radiation therapy.

With the advent of intensity‐modulated radiation therapy (IMRT) and volumetric modulated arc therapy (VMAT), three‐dimensional LRT can now be delivered using a variety of vertex arrangements to create the desired peak‐and‐valley dose distribution. This advancement enables the highly conformal delivery of high‐dose vertices, thereby offering superior protection for the skin and surrounding normal tissues compared to earlier techniques. Currently, vertices in LRT can be configured either manually or through automated generation. While spherical vertices are most common, cylindrical shapes have also been investigated. In manual placement, clinicians typically utilize basic geometric tools within the treatment planning system, such as distance measurement and 3D visualization, to position vertices according to specific institutional protocols.^8^, ^9^ For instance, Borzov et al.^10^ applied LRT to large soft‐tissue sarcomas by manually placing high‐dose cylinders (1 cm in diameter and height) within the tumor volume, with their number and location determined by the tumor size and proximity to organs at risk. Duriseti et al.^11^ proposed a method of placing spherical vertices at the centroid of the GTV (Gross Tumor Volume). Lattice sphere centers were placed in a 5 mm isotropic erosion of the GTV. This method employs vertices with a 1.5 cm diameter sphere in a face‐centered cubic (FCC) close‐packing with a center‐to‐center spacing of 3√2 cm to the nearest neighboring sphere. In addition, the lattice sphere edges must also be at least 1.0 cm from any nearby critical organs, and any part of the lattice sphere outside the GTV must be removed.

While existing methods allow for the manual placement of vertices, this process can be laborious and highly subjective, particularly for large or irregularly shaped tumors, with no guarantee of optimal vertex configuration. To address these limitations, research into automated vertex generation and optimization has emerged. In 2024, Tucker et al.^12^ contributed significantly to standardizing the procedure by developing a C# script for automated vertex generation in LRT. Their rule‐based algorithm defined the following parameters: high‐dose spheres (HDS), serving as vertices, were 1.5 cm in diameter and 3√2 cm in center‐to‐center spacing, collectively forming a FCC close‐packing geometry. A critical constraint required all HDS to be entirely contained within a retracted GTV, with the retraction margin adjustable based on the proximity of organs at risk. The entire grid was permitted to translate in 3D space and rotate about the craniocaudal axis in 10° increments up to 90° to maximize the number of HDS within the retracted volume. HDS were allowed a translational offset of up to 3 mm in any direction to better conform to the GTV shape or avoid OARs. However, a notable limitation of this approach is that the optimization process did not modulate key geometric parameters such as vertex diameter and center‐to‐center distance, consequently failing to ensure the maximization of the peak‐to‐valley dose ratio.

In 2025, Ma et al.^8^ introduced a methodology that utilizes hexagonal close‐packing (HCP) to generate initial LRT vertex positions, which are subsequently optimized via an adaptive simulated annealing (ASA) algorithm to refine center‐to‐center distance and distribution, with the explicit objective of maximizing the peak‐to‐valley dose ratio. Their vertex placement protocol was as follows: the initial vertex coordinates were defined according to an HCP lattice, where the starting spacing and diameter were determined as functions of the GTV. This initial configuration was then input into the ASA algorithm for optimization. The primary objective function was to maximize the distances between vertices, thereby enhancing the peak‐to‐valley dose ratio. The optimization was constrained by two key conditions: all vertices were required to remain within an inwardly contracted margin of the GTV, and the total volume of all vertices was maintained constant at 1% of the GTV. A critical distinction from the method proposed by Tucker et al. is that this approach allows for rotational adjustments in three dimensions, resulting in a significantly larger solution space for identifying the optimal configuration. The results demonstrated that the optimized plans achieved an approximately five‐fold increase in the peak‐to‐valley index (PVI) compared to the non‐optimized HCP baseline.

Close‐packing in crystallography refers to the arrangement of atoms or molecules that achieves the highest possible packing density within a given volume. The two fundamental types of close‐packing are HCP and FCC, both of which attain an identical maximum spatial occupancy of approximately 74.05%.^13^ In the context of LRT, close‐packing is leveraged to position the maximum number of spherical vertices within a defined target volume. While both HCP and FCC methods have been independently employed for LRT vertex placement, a direct comparative analysis of these two specific geometric configurations is currently absent from the literature. This study aims to systematically compare these two close‐packing methods for LRT vertex arrangement under identical conditions, to determine which method yields superior dosimetric outcomes and facilitates the establishment of standardized guidelines for LRT planning.

### 1.1 Materials and methods

This study was retrospectively conducted on 10 patients with large gross tumor volumes who previously underwent radiation therapy at the National Cancer Center/Cancer Hospital, Chinese Academy of Medical Sciences. The GTV and critical structures were contoured and reviewed during a chart round by radiation oncologists. The average GTV was 1277.30 ± 1069.38 cm^3^ (range, 319.24–4116.82 cm^3^).

### 1.2 Vertex placement

For each patient in the study cohort, two distinct LRT plans were generated: Plan_HCP and Plan_FCC. These plans were differentiated by the geometric arrangement of vertices, which followed the principles of HCP and FCC, respectively, as illustrated in Figure 2. Subsequently, the vertex positions for both plans were optimized to maximize the center‐to‐center distance, thereby enhancing the peak‐to‐valley dose ratio. To ensure a dosimetrically fair comparison, both plans utilized spherical vertices of identical size and shape. The number of vertices was kept comparable between the two plans, with the total volume of all vertices constrained to be approximately 1% of the GTV volume. The vertex placement workflow consisted of three sequential stages: 1) generation of the lattice volume, 2) initial placement of vertices according to the HCP or FCC methods, and 3) optimization of final vertex positions.

**FIGURE 2:**
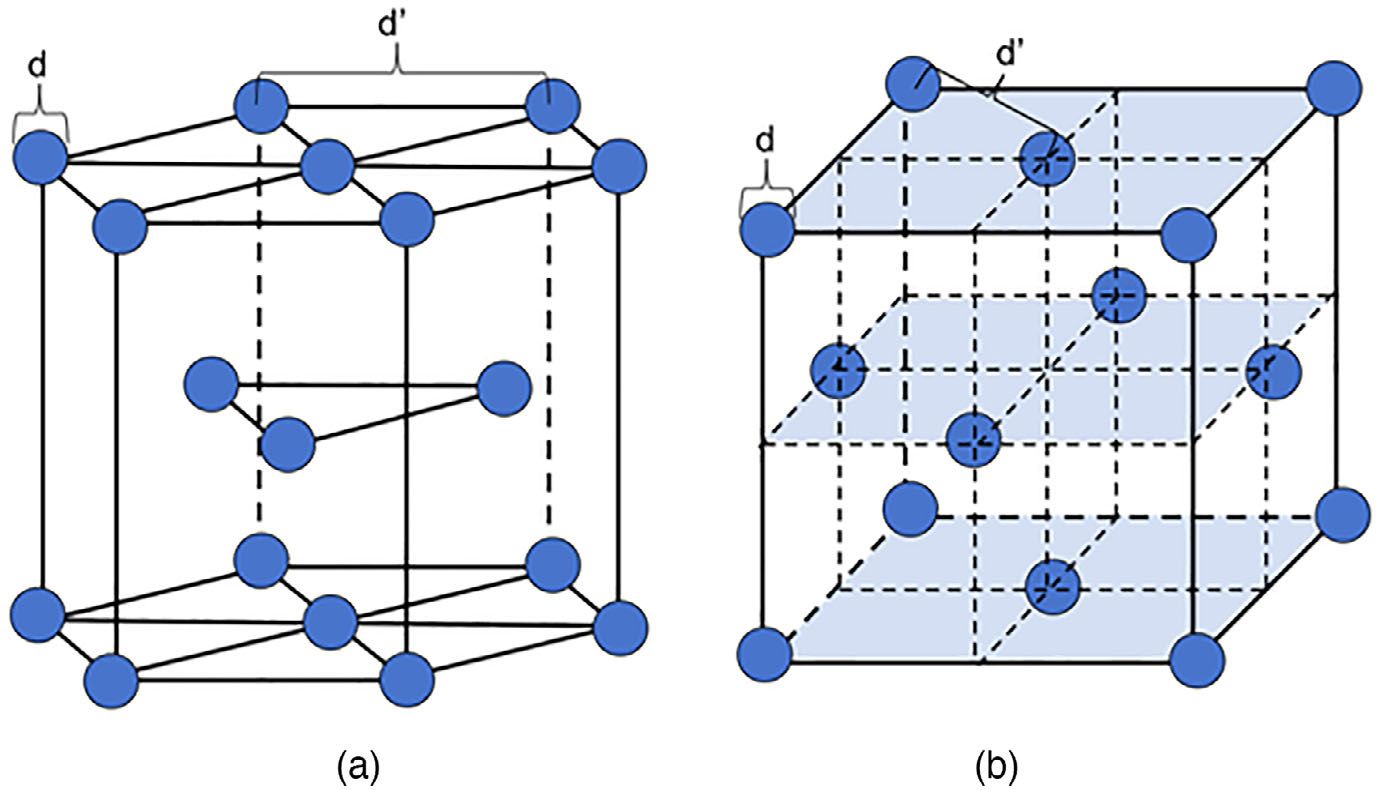
Schematic diagram of the spatial arrangement of vertices. (a) Hexagonal close‐packed (HCP) lattice. (b)Face‐centered cubic (FCC) lattice. The diameter d and the center‐to‐center distance $d^{\prime }$ of vertices were defined.

#### 1.2.1 Lattice volume for vertex arrangement

Given the high dose prescription to the vertices, maintaining a sufficient distance from organs at risk (OARs) was imperative. The spatial relationship between the tumor and surrounding normal tissues was analyzed based on patient CT images and the contours of the GTV and OARs to define the lattice volume.^7^ This volume was generated by applying an isotropic inward margin to the GTV. The magnitude of this inward contraction (d_inward_) was determined according to the formula established in Reference, 8 which was itself derived from fitting clinical data presented in Reference:^7^

(1) $$d_{inward}=\left\{\begin{array}{cc} 1, & V_{GTV}<200cm^{3}\\ 0.00125V_{GTV}+0.75, & 200\leq V_{GTV}<1000cm^{3}\\ 2, & V_{GTV}\geq 1000cm^{3} \end{array}\right.$$

where V_GTV_ represents the volume of the patient's GTV.

#### 1.2.2 Initial vertex placement

The geometric center of the GTV was selected as the coordinate for the first vertex center. Subsequently, the positions of all remaining vertex centers were algorithmically generated according to the distinct spatial periodicity of either HCP or FCC. This initial arrangement was designed to ensure comprehensive coverage of the GTV volume. The initial diameter and the center‐to‐center distance of vertices were defined as functions of the GTV. The diameter of vertices *d* was specifically calculated using Equation (2), as described in References 8 and 7:

(2) $$d=\left\{\begin{array}{cc} 0.5\;cm, & V_{GTV}<50\;cm^{3}\\ 0.001V_{GTV}+0.4258\;cm, & 50\leq V_{GTV}<1000\;cm^{3}\\ 1.5\;cm, & V_{GTV}\geq 1000\;cm^{3} \end{array}\right.$$

The initial center‐to‐center distance of vertices $d^{\prime }$ was derived from Equation (3) as referenced in 7 and 8:

(3) $$d^{\prime }=\left\{\begin{array}{cc} 2\;cm, & V_{GTV}<50\;\;cm^{3},\\ 0.002V_{GTV}+1.9\;cm, & 50\leq V_{GTV}<1000\;\;cm^{3},\\ 4\;cm, & V_{GTV}\geq 1000\;\;cm^{3}. \end{array}\right.$$

where V_GTV_ represents the volume of the patient's GTV.

#### 1.2.3 Optimization of final vertex positions

Adaptive simulated annealing (ASA) was employed to optimize vertex positions in LRT planning.^8^ This algorithm offers several distinct advantages over conventional simulated annealing: it permits independent cooling schedules for different parameters, enhancing computational efficiency; it incorporates a reannealing feature that adaptively refines the search sensitivity when navigating the solution space; and it provides extensive tunability with over 100 options for complex nonlinear stochastic systems.^14^

Leveraging these capabilities and its public availability, ASA was used to iteratively adjust vertex positions through translations and rotations. This process maintained a constant total vertex volume with the explicit objective of maximizing center‐to‐center distances, thereby amplifying the peak‐to‐valley dose difference. The optimization process was constrained by the requirement that the total volume of all vertices must constitute at least 1% of the GTV and any part of the lattice sphere outside the lattice volume will be removed. If the resulting total volume fell below the 1% GTV threshold, the diameters of vertices in both planning approaches were systematically adjusted upwards until the prescribed volume ratio was satisfied.

### 1.3 LRT treatment planning

Treatment planning was performed using the Pinnacle^3^ treatment planning system (version 9.16; Philips Healthcare, Andover, MA, USA). For tumors with a length less than 20 cm, plans were designed using 6 MV Flattening Filter‐Free (FFF) photon beams and were intended for delivery on a Varian Edge linear accelerator (Varian Medical Systems, Palo Alto, CA, USA) equipped with a 60 pair multi‐leaf collimator (MLC). The central 10 cm of the MLC had a leaf width of 2.5 mm, while the remaining leaves had a width of 5 mm. For tumors longer than 20 cm, 6 MV FFF beams were also used, with plans designed for delivery on an Elekta VersaHD linear accelerator equipped with an 80 pair MLC, all leaves of which had a width of 5 mm. The dose calculation grid resolution was set to 0.2 × 0.2 × 0.2 cm^3^. Between 3 and 5 coplanar partial arcs were used, with the exact number determined based on the geometric complexity of the tumor.

The same dose–volume constraints were applied to all the plans during inverse planning optimization. The final dose distributions were calculated using adaptive convolution. The planning objective was to deliver the prescription dose of 60 Gy to at least 95% of the volume of the vertices in 4 fractions. To enforce rapid dose fall‐off, three concentric ring structures were generated around each vertex: Ring 1 at a 5 mm distance from the vertex boundary, Ring 2 at 10 mm, and Ring 3 at 15 mm. To enhance the dose gradient, additional spheres between the vertices were created to reduce the dose in the valley region. These auxiliary low‑dose spheres were placed at the midpoint of the line segment connecting two neighboring vertices. Furthermore, patient‐specific optimization was performed to minimize the dose to normal tissues outside the GTV, while achieving the prescription dose and keeping the valley dose below 5 Gy. The optimization for each individual case aimed to ensure the delivery of the prescribed peak dose while minimize the dose to the valley and the normal tissue outside the GTV.

### 1.4 LRT plan evaluation

To evaluate plan quality, this study adopted the dose metrics for the GTV recommended by the Radiosurgery Society SFRT working group and the AAPM Task Group 440, including D_5_%, D_10_%, D_50_%, D_90_%, D_mean_, V_50_%, and the peak‐to‐valley dose ratio (PVDR = D_10_% ÷ D_90_%).^15^, ^16^ However, since vertices are confined within the lattice volume, the D_90_% of the GTV primarily reflects the dose at the tumor periphery and may not accurately represent the dose heterogeneity influenced by the vertex distribution. Therefore, the D_10_/D_90_ ratio for the lattice volume—defined as the ratio of the doses covering 10% (D_10_) and 90% (D_90_) of the lattice volume—was also calculated and analyzed in this study. Furthermore, the mean dose of normal tissue (NT) was then used to assess toxicity in all normal tissues NT was defined on each slice by subtracting both the gross tumor volume (GTV) from the patients contour.

## 2 RESULTS

### 2.1 Vertex arrangement

Table 1 presents the results of two different vertex arrangement methods, including the number, the volume and the center‐to‐center distance of vertices. With identical vertex diameters, the number, volume, and spacing of vertices in both Plan_FCC and Plan_HCP followed a normal distribution. Wilcoxon signed‑rank test indicated no statistically significant differences (*P* > 0.05) in vertex number, volume, or spacing between the two arrangements, thereby ensuring that the dosimetric comparisons were not confounded by factors other than the geometric packing configuration itself. Especially, no statistically significant difference was observed in the center‑to‑center distance between FCC and HCP plans (*p* = 0.081), confirming that the dosimetric comparison was not biased by spacing differences.

**TABLE 1:** Lattice deployment metrics comparison between HCP and FCC.

	Vertex arrangement		*P*‐Value (*α* = 0.05)
	HCP	FCC	
GTV(cm^3^)	916.59[609.37]		
The lattice volume(cm^3^)	297.56[187.81]		
Diameter of vertices(mm)	13.95[2.08]		
Number of vertices	9[2.50]	9.5[1.75]	0.638
Volume of vertices(cm^3^)	9.28[6.47]	9.32[5,96]	0.831
Center‐to‐center distance (mm)	38.9[7.15]	38.8[6.12]	0.081
Volume ratio (Vertices/GTV,%)	1.03[0.00]	1.01[0.00]	0.894

*Note*: Data are presented as median [IQR], where IQR = Q3 − Q1.

Abbreviations: FCC: face‐centered cubic; GTV: gross tumor volume; HCP: hexagonal close‐packed.

### 2.2 Dosimetry data

Table 2 provides the extracted GTV and NT dose metrics from plans using contours from HCP and FCC. Table 3 presents the extracted D_max_ values for site‐specific organs. No significant differences were observed between HCP and FCC for any of the evaluated OARs (*p* > 0.05). Plan_FCC was able to demonstrate a statistically significant increase in PVDR(lattice volume D_10_/D_90_) (Δmean = 1.64, p < 0.05). However, no significant differences were observed in the other evaluated metrics.

**TABLE 2:** Dose metrics from two plans where lattice contours were derived using either HCP or FCC.

Dose Metric	Vertex Arrangement		*P*‐Value (*α* = 0.05)
	HCP	FCC	
GTV D_5_ (Gy)	38.06[3.91]	38.08[6.31]	0.447
GTV D_10_ (Gy)	28.98[4.28]	28.88[5.53]	0.749
GTV D_50_ (Gy)	13.03[4.85]	11.94[5.81]	0.577
GTV D_90_ (Gy)	3.36[1.61]	2.52[2.27]	0.204
GTV D_mean_ (Gy)	14.44[3.15]	13.93[4.37]	0.760
Lattice volume D10	42.93[7.58]	44.42[6.88]	0.082
Lattice volume D90	9.30[4.25]	6.93[4.69]	0.095
PVDR(GTV D_10_/D_90_)	8.28[3.61]	11.17[16.04]	0.203
PVDR(lattice volume D_10_/D_90_)	4.48[2.00]	6.95[3.38]	**0.019**
NT D_mean_ (Gy)	3.15[1.10]	2.99[1.50]	0.334

*Note*: Data are presented as median [IQR], where IQR = Q3−Q1.

Abbreviations: FCC: face‐centered cubic; GTV: gross tumor volume; HCP: hexagonal close‐packed; NT: normal tissue.

**TABLE 3:** Extracted D_max_ values for site‐specific organ.

Treatment site	number	OAR evaluated	Sphere arrangement		*P*‐Value
			HCP	FCC	
			D_max_(Median [IQR])		
Abdominal/Pelvis	5	Intestine	19.42[3.15]	12.66[8.81]	0.365
		Colon	24.72[11.67]	30.49[8.85]	0.203
		Cord	6.49[10.40]	8.52[15.90]	0.216
Thigh	5	Bone	22.83[1.6]	20.45[0.91]	0.442

*Note*: Data are presented as median [IQR], where IQR = Q3−Q1.

### 2.3 Representative patient

Figure 3 displays dose distribution for a representative patient, and Figure 4 shows the corresponding DVH comparison. Owing to the different vertex distribution planes among the three plans, as well as the translation and rotation applied during optimization, a direct comparison of the corresponding layers was not possible. Therefore, only representative layers are depicted. The patient's GTV measured 543.63 cm^3^, with a lattice volume of 135.99 cm^3^. The vertex volumes in Plan_HCP and Plan_FCC were 5.67 cm^3^ (6 vertices) and 5.51 cm^3^ (6 vertices), respectively. The center‐to‐center distances of vertices were 34.9 mm for Plan_HCP and 34.9 mm for Plan_FCC, with both plans employing a vertex diameter of 14 mm.

**FIGURE 3:**
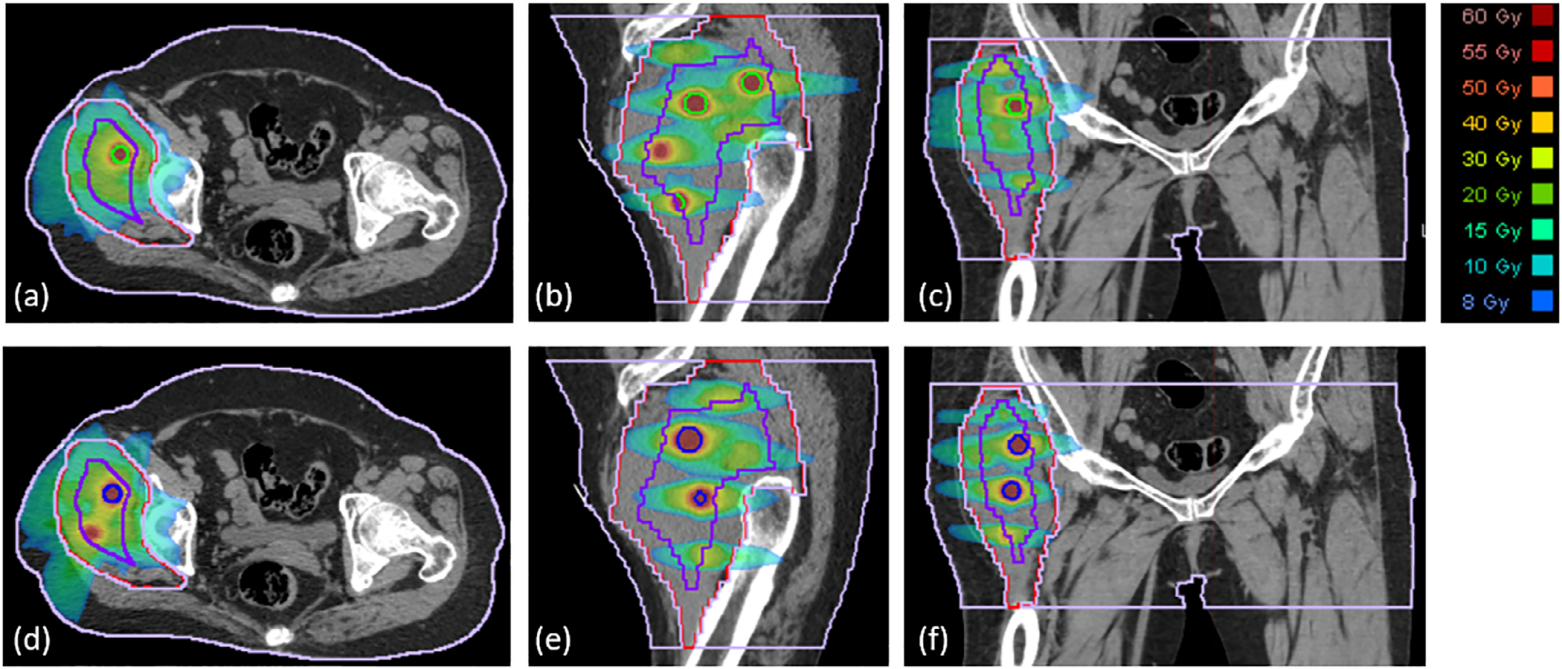
Vertex placement and dose distribution for Hexagonal Close‐Packing (HCP) and Face‐Centered Cubic (FCC) arrangements. Panels (a)‐(c) illustrate the resulting lattice plan using the HCP method (green spheres, *n* = 6). Panels (d)‐(f) show the corresponding plan using the FCC method (blue spheres, *n* = 6). The gross tumor volume (GTV) is outlined in red, and nearby normal tissue (NT) is contoured in lavender. The lattice volume is shaded in purple. An isodose color wash, ranging from 8 to 60 Gy, depicts the dose distribution.

**FIGURE 4:**
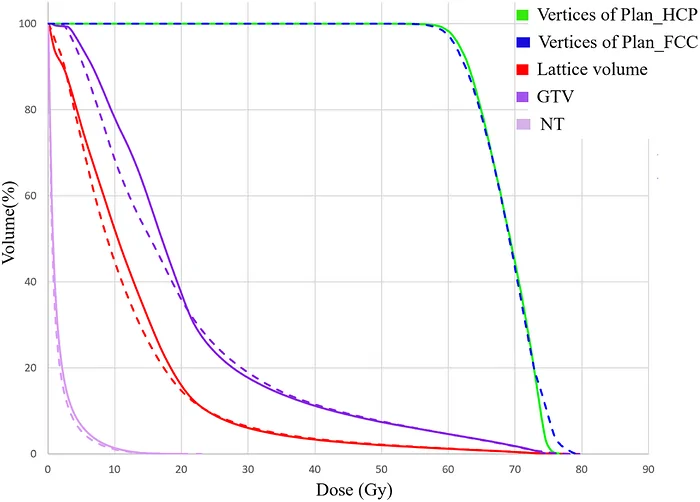
Dose‐volume histogram (DVH) for Hexagonal Close‐Packing (HCP) and Face‐Centered Cubic (FCC) arrangements for a representative patient.

Dosimetric evaluation revealed that Plan_FCC achieved a superior peak‐to‐valley dose ratio (PVDR, defined as lattice volume D_10_/D_90_) compared to Plan_HCP, with values of 8.09 and 6.44, respectively. Key dose‐volume metrics for the GTV, extracted from the differential DVH, are as follows: for Plan_HCP, D_5_%, D_10_%, D_50_%, and D_90_% were 32.86 Gy, 23.92 Gy, 10.46 Gy, and 2.26 Gy, respectively; corresponding values for Plan_FCC were 33.73 Gy, 23.99 Gy, 8.84 Gy, and 2.46 Gy. The mean dose to the GTV (D_mean_) was 14.69 Gy for Plan_HCP and 14.77 Gy for Plan_FCC. The volume of the GTV receiving at least 50% of the prescription dose (V_50_%) measured 32.7 cm^3^ for Plan_HCP and 34.35 cm^3^ for Plan_FCC. Regarding normal tissue sparing, the mean dose to normal tissue (NT D_mean_) was 1.50 Gy for Plan_HCP and 1.31 Gy for Plan_FCC.

## 3 DISCUSSION

Our study aimed to compare two close‐packing vertex arrangements during LRT planning for managing large and bulky tumors. A statistically significant difference was observed in the PVDR (lattice volume D_10_/D_90_) between Plan_HCP and Plan_FCC, whereas no significant differences were found in other dosimetric indices. This discrepancy may be attributed to the distinct geometric symmetries of the two arrangements. From the perspective of geometric voids, both HCP and FCC lattices contain tetrahedral and octahedral voids. Notably, the octahedral voids are aligned along consistent orientations in the FCC structure, whereas their arrangement varies in direction within the HCP lattice; the pattern of the “valleys” still conforms to a close‐packed arrangement in FCC, but not in HCP, as illustrated in Figure 5. This difference in void orientation may affect the dose distribution in the regions between vertices inside the lattice volume, potentially affecting the D_90_ value within the lattice volume.

**FIGURE 5:**
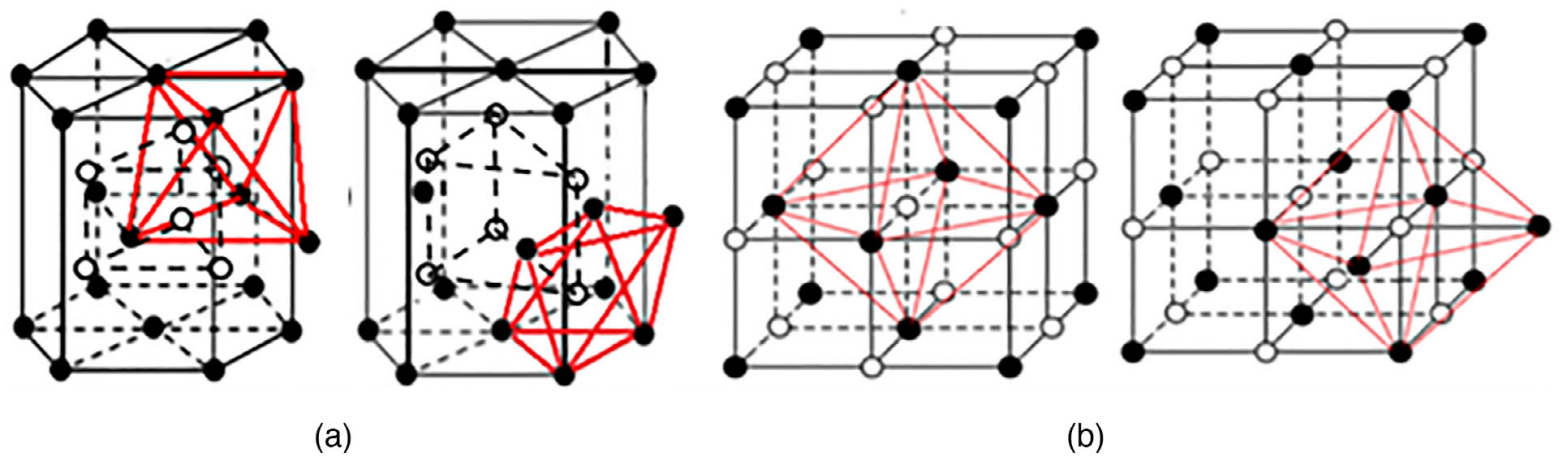
Schematic diagrams illustrating geometric differences between HCP and FCC. (a) Octahedral void in a hexagonal close‐packed (HCP) lattice. (b) Octahedral void in a face‐centered cubic (FCC) lattice. Black spheres represent the vertices, the octahedron outlined by red lines represents the void; white spheres represent the center of the octahedron, which corresponds to the valley.

Beyond quantitative metrics, qualitative examination of isodose lines revealed that for FCC, valley dose isodose lines between adjacent vertices remained mostly separated, whereas HCP occasionally exhibited “bridges” of dose between vertices. It can be seen in Figure 3 that, compared with panels (e) and (f), panels (b) and (c) show higher valley doses that appear to connect the vertices together. This spatial separation may contribute to the higher PVDR of FCC. More importantly, the absolute valley dose is clinically more relevant than PVDR alone. As suggested by Wu et al.,^15^ valley dose should be kept below 5 Gy to preserve circulatory function. In our plans, both configurations achieved valley doses < 5 Gy in all patients (median HCP: 3.36[1.62] Gy; FCC: 2.52[2.28] Gy;). Thus, while a higher PVDR is desirable, it should not be used without verifying the absolute valley dose.

However, no universally standardized methodology currently exists for calculating the PVDR. Some studies alternatively employ its reciprocal, the valley‐to‐peak dose ratio (VPDR), as an evaluation parameter. Wu et al.^17^ defined a specific VPDR method (VPDR_Wu), where the peak dose is defined as the prescription dose, and the valley dose is defined as the mean dose to the region receiving the lowest 5% of dose within the lattice target volume (D_avg(95∼100)). As the valley dose is derived from a relatively large area, this method may potentially overestimate the valley dose. Conversely, Prado et al.^18^ proposed the VPDR_Median method, defining the peak dose as the D1% dose (i.e., the minimum dose covering 1% of the vertex volume) for each pair of adjacent vertices, and the valley dose as the dose at the midpoint between them. The valley‐to‐peak dose ratio is calculated for each vertex pair, and the median of these ratios is reported. In the study by Tucker et al.,^12^ the peak‐to‐valley dose ratio (PVDR) was defined as the ratio of the mean dose to high‐dose spheres (HDS) and the mean dose to low‐dose spheres (LDS). Ma et al.^8^ introduced a newly defined metric, the peak‐to‐valley index (PVI), to characterize the dose heterogeneity. Overall, different methods of calculating the PVDR focus on distinct aspects of dose distribution. Overall, different methods of calculating the PVDR focus on distinct aspects of dose distribution. We acknowledge that our chosen metric (D10/D90) also has inherent limitations. In our study, we used D10/D90 for both GTV and lattice volume. However, as noted by Prado et al.,^18^ D10/D90 may not properly describe the ‘true’ valley and peak doses. Specifically, when the vertex volume comprises only ∼1% of the GTV, the D10% of the GTV or lattice volume underestimates the true peak dose, while the D90% overestimates the true valley dose. Despite this, a difference in lattice volume D10/D90 was observed between the HCP and FCC methods, no significant difference was found in GTV D10/D90. This may be attributed to the fact that vertices are distributed only within the lattice volume, so the D10 and D90 within the lattice volume are influenced by the vertex arrangement. In contrast, GTV D90 is more affected by the lower doses in the eroded marginal region of the GTV. When vertex size and total volume are fixed, the difference in lattice volume D10/D90 suggests that the two arrangement methods may lead to greater dose heterogeneity between peaks and valleys inside the lattice volume, reflecting a higher degree of dose distribution inhomogeneity. These methodological variations impede direct comparison of PVDR/VPDR values across studies, and the generalizability of our clinical findings warrants further robust validation.

In addition to arranging LRT vertices according to close‑packed geometries, multiple studies have investigated alternative methods to optimize sphere packing and placement, as well as to automate this process. For example, Deufel et al.^19^ proposed a Monte Carlo‑based approach in which the algorithm attempts 100,000 trials to pack N spheres and then iteratively repeats the process for N+1 spheres until no further solution can be found. Similarly, Misa et al.^20^ introduced an algorithm utilizing the Rödl nibble methodology to optimize the number and arrangement of lattice vertices for spatially fractionated radiotherapy planning. While the close‑packed arrangement employed in our study achieves the highest spatial filling efficiency, it may not be optimal for irregularly shaped tumor volumes. A strictly periodic configuration might not accommodate the maximum number of spheres, and even with rotation or translation of HCP or FCC lattices, the fit to the actual lattice volume boundary can remain suboptimal. In future work, advanced optimization algorithms can be developed to refine vertex placement near the volume periphery, enhancing both geometric conformity and dosimetric coverage.

Accumulating evidence indicates that tumors exhibit significant heterogeneity, leading to high variability in dose response across different regions, which can drastically impact clinical outcomes.^21^, ^22^ Generating spatially heterogeneous dose distributions that account for intra‐tumor dose response variability holds considerable clinical significance, a concept known as metabolic‐guided LRT.^23^ Tubin et al.^24^ proposed a technique called Stereotactic Body Radiation Therapy for Partial Tumor Irradiation Targeting the Hypoxic Segment of Bulky Tumors (SBRT‐PATHY). This approach utilizes fluorodeoxyglucose positron emission tomography (FDG‐PET) and contrast‐enhanced CT to identify the tumor location and assess the metabolic activity and perfusion status of its various sub‐regions. FDG‐PET imaging helps localize hypoxic areas within the tumor. Furthermore, Yuan et al. proposed and validated a biology‐guided LRT (BG‐LRT) strategy based on Apparent Diffusion Coefficient (ADC) maps, which precisely positions high‐dose lattice vertices within regions exhibiting low ADC values to improve tumor control probability while sparing normal tissues. Future refinements could integrate such clinical information by assigning high weights during optimization to ensure LRT vertices are strategically positioned within metabolically active or radioresistant sub‐volumes. The present study has several strengths, including a systematic, geometry‑driven comparison of two close‑packing vertex arrangements, a PVDR evaluation explicitly confined to the lattice volume (rather than the entire GTV), and a reproducible methodology that can be readily implemented in other treatment planning systems.

This study has several limitations. First, the sample size is small (*n* = 10), and all patients were treated at a single institution, which limits the generalizability of our findings. Second, no clinical outcomes (e.g., local control, toxicity, survival) were reported; the dosimetric superiority of FCC in terms of PVDR does not necessarily translate into improved clinical benefit. Third, our results were obtained using a 6 MV flattening‑filter‑free (FFF) beam and designed for delivery on two linear accelerator models (Varian Edge and Elekta VersaHD) with different MLC configurations. The observed differences between FCC and HCP may vary with beam energy, MLC resolution, delivery technique (VMAT vs. fixed‑field), or fractionation scheme. Furthermore, we used coplanar arcs for all plans, which may limit the ability to separate valley doses. Fourth, the ASA optimization algorithm performed only rigid translation and rotation of the entire vertex lattice; local adjustments near the GTV boundary were not applied, which may have left some geometric conformity unoptimized.

Future research directions include: (1) multicenter studies with larger patient cohorts to validate the dosimetric benefits of FCC; (2) integration of clinical outcome data to establish dose‑response relationships for lattice radiotherapy; (3) biology‑guided vertex placement (as discussed above) using functional imaging (FDG‑PET, ADC maps) to target radioresistant sub‑volumes; (4) investigation of non‑coplanar beam arrangements, which may further improve valley dose separation by altering the incident beam angles relative to the vertex lattice; and (5) development of advanced optimization algorithms that allow vertex positions to deform adaptively to irregular tumor boundaries while preserving close‑packing efficiency.

## 4 CONCLUSION

This study generated and compared two LRT plans for each patient by arranging vertices according to FCC and HCP geometries, followed by vertex position optimization using the Adaptive Simulated Annealing (ASA) algorithm to maximize center‐to‐center distance. Under otherwise identical planning parameters, the impact of these two close‐packing arrangement methods on plan quality was systematically evaluated. The results indicate that, under the evaluated planning conditions, the FCC configuration can increase the PVDR (lattice‐volume D_10_/D_90_), with statistical significance, while no statistically significant difference in mean normal‐tissue dose was observed, suggesting that FCC may be considered as an initial configuration for LRT vertex placement in large tumors, although its clinical relevance requires validation in larger prospective studies.

## AUTHOR CONTRIBUTIONS

**Jianrong Dai**: Conceptualization, formal analysis, investigation, methodology, project administration, writing–review and editing. **Pan Ma**: Data curation, project administration, resources, supervision, writing–review and editing. **Yingjie Song**: Conceptualization, investigation, formal analysis, methodology, resources, software, validation, writing–original draft.

## FUNDING INFORMATION

The author(s) declare financial support was received for the research and/or publication of this article. This work was supported by the National Natural Science Foundation of China (Grant No. 11875320).

## CONFLICT OF INTEREST STATEMENT

The authors declare no conflicts of interest.

## ETHICAL APPROVAL

All procedures were carried out in accordance with the Declaration of Helsinki. This retrospective study was approved by the Institutional Review Board (IRB) of National Cancer Center/Cancer Hospital, Chinese Academy of Medical Sciences and Peking Union Medical College. Patient consent was waived due to the retrospective nature of the study.

## Data Availability

Data are available from the corresponding author on reasonable request.
